# Safety and efficacy of cataract surgery performed with a low-energy femtosecond laser compared with conventional phacoemulsification in Chinese patients: a randomized clinical trial

**DOI:** 10.1186/s40662-023-00347-0

**Published:** 2023-07-02

**Authors:** Kai-Jing Zhou, Yusen Huang, Yong Wang, An-Peng Pan, Xu Shao, Rui-Xue Tu, A-Yong Yu

**Affiliations:** 1 National Clinical Research Center for Ocular Diseases, Eye Hospital, Wenzhou Medical University, 270 West Xueyuan Road, Wenzhou, 325000 Zhejiang China; 2 Qingdao Eye Hospital of Shandong First Medical University, Shandong, China; 3 Aier Eye Hospital of Wuhan University, Wuhan, China

**Keywords:** Cataract surgery, Femtosecond laser-assisted cataract surgery, Low-energy FLACS, FLACS versus manual, FEMTO LDV Z8

## Abstract

**Background:**

To compare the safety and efficacy of femtosecond laser-assisted cataract surgery (FLACS) performed with the low-energy FEMTO LDV Z8 (Ziemer Ophthalmic Systems AG, Port, Switzerland) laser compared with conventional phacoemulsification (CP) in Chinese patients.

**Methods:**

This prospective, multicenter, interventional study included 126 patients who were randomized (1:1) to undergo either FLACS or CP followed by intraocular lens (IOL) implantation between January 2019 and April 2020. The primary endpoint included the comparison of the endothelial cell loss (ECL) between the two groups at 3 months. Secondary endpoints included the comparison of cumulative dissipated energy (CDE), change in central corneal thickness (CCT) from baseline, and postoperative uncorrected and corrected distance visual acuities (UDVA and CDVA) in the two groups.

**Results:**

At all postoperative time points, the FLACS group was found to be non-inferior to CP for the mean ECL (− 409.3 versus − 436.9 cells/mm^2^ at 3 months) and mean CDE (4.1 versus 4.5 percent-seconds). The increase in CCT was significantly lower in the FLACS group compared with the CP group at Day 7 (4.9 versus 9.2 µm; *P* = 0.04); however, the difference was not statistically significant at 1 and 3 months. Postoperatively, mean UDVA and CDVA were comparable between the two groups. No intraoperative complications occurred.

**Conclusions:**

Cataract surgery performed with a low-energy femtosecond laser was non-inferior to CP; however, the FLACS group had a statistically significantly lower increase in CCT at Day 7 compared with CP.

*Trial registration* This trial is registered at ClinicalTrials.gov on May 15, 2019, with trial registration number: NCT03953053.

**Supplementary Information:**

The online version contains supplementary material available at 10.1186/s40662-023-00347-0.

## Background

Cataract surgery is the most commonly performed ophthalmic procedure worldwide. Although phacoemulsification is effective in providing good visual acuity, the dissipation of ultrasonic energy during phacoemulsification causes mechanical and thermal damage to the corneal endothelium [1]. Corneal endothelial cells play a pivotal role in maintaining corneal transparency; therefore, damage to corneal endothelium function may lead to corneal edema and, in advanced stages, corneal decompensation/bullous keratopathy [2]. As such, precise corneal thickness measurements may serve as an important metric for assessing overall corneal endothelium function.

In recent years, femtosecond lasers have gained popularity and are being used to assist in important steps of cataract surgery, including corneal incisions, capsulotomy, and lens fragmentation [3]. Femtosecond laser-assisted cataract surgery (FLACS) has been found to reduce the phacoemulsification time and energy, minimize collateral tissue damage and reduce anterior chamber manipulation, thereby limiting ECL and reducing anterior chamber inflammation [4–6]. This may translate into quicker recovery and improved cataract surgery outcomes [7, 8].

Although advantageous, higher cost, longer operating times, and the need for an additional operating area restrict the wide adoption of FLACS. The need to shuttle patients between rooms to complete surgery not only adds time but also increases the risk of infection [9]. In some studies, the risk of complications such as incomplete capsulotomies, anterior capsulotomy tags, and anterior capsular tears have been found to be higher with FLACS [10–13]. As such, femtosecond laser systems that can overcome the above issues are desirable.

FEMTO LDV Z8 (Ziemer Ophthalmic Systems AG, Port, Switzerland) is a versatile mobile laser platform with a small clinical footprint that fits in a small space and allows surgery to be completed in a single room without the need to move the patient or the bed resulting in overall lower operating time [14, 15]. It employs the concept of overlapping low-energy femtosecond laser pulses in the nano-Joule range and operates at a high frequency achieving a repetition rate in the MHz range and creating consistent, circular and smooth capsulotomies through clear corneas, with minimal release of inflammatory mediators and no significant pupillary constriction [16–19].

The present study was aimed at evaluating the safety and efficacy of low-energy FEMTO LDV Z8 laser-assisted cataract surgery in comparison with conventional phacoemulsification (CP) in the Chinese population.

## Methods

This prospective, multi-center, interventional, randomized controlled trial (registration number: NCT03953053) included cataract patients who were randomized to undergo either low-energy FLACS or CP followed by intraocular lens (IOL) implantation between January 2019 and April 2020 at three clinical centers in China (Wenzhou Eye Hospital, Wenzhou; Qingdao Eye Hospital, Qingdao and Wuhan Aier Hospital, Wuhan). The study was approved by the Ethics Committees of the respective investigational sites (reference number: 2018-8-Q-6) and adhered to the tenets of the Declaration of Helsinki. The study followed the requirements of the “Medical Devices Registration Administration Method” issued by the National Medical Products Administration (NMPA), Medical Device GCP issued by the NMPA and Chinese National Health and Family Planning Commission (NHFPC). Written informed consent was obtained from all patients prior to participation.

### Recruitment criteria

The inclusion criteria included males or females aged between 50 and 80 years, who were scheduled to undergo cataract surgery with the implantation of a monofocal aspheric IOL. Patients who provided written informed consent and who were willing to comply with all study procedures and return for scheduled follow-up examinations were included. Only one eye per subject was included in the study. Patients were randomly assigned in a 1:1 ratio to receive either FLACS with low-energy FEMTO LDV Z8 laser or CP. Randomization was performed using sequentially numbered, opaque, sealed envelopes. The sequence in which participants were allocated to treatment had been generated with a computerized random number generator. To ensure allocation concealment, the investigators received sequentially numbered, opaque, sealed envelopes to prevent patients and investigators from knowing the treatment allocation before randomization. Treatment allocation was revealed only after patients had been enrolled. Decentralized randomization (random grouping) was performed in each clinical center to ensure that an equal number of patients were randomized to the two treatment groups in each center. In the case of bilateral cataracts, the treatment eye was specified in the randomization list.

Patients were excluded if they had any of the following in the study eye: corneal disease or corneal endothelial pathology; poorly dilating pupil or other pupillary defects; glaucoma, hypotony or ocular hypertension, pseudoexfoliation; complicated cataract, such as traumatic, white, intumescent or posterior polar cataract and anterior subcapsular cataract; zonular instability; keratoconus or keratectasia; anterior chamber depth < 1.5 mm or > 4.8 mm. Additional exclusion criteria included any previous intraocular or corneal surgery; nystagmus or hemifacial spasm preventing placement of the patient interface; allergy to any pre/perioperative medications; acute or chronic illnesses that in the opinion of the principal investigator of the site could possibly increase the risk to the subject or confound the outcomes of this study; developmental disability or cognitive impairment that would make informed consent and the assessment of visual acuity impossible; and concurrent participation in another ophthalmological clinical study.

### Study procedures

All eligible patients underwent standard preoperative examination. Cataract density grading for each eye was performed using a Scheimpflug imaging device (Pentacam HR; Oculus, Wetzlar, Germany). Prior to the surgery, all patients were prescribed topical antibiotic and non-steroidal anti-inflammatory eye drops for 2–3 days. Pupil dilation was achieved with 0.5% tropicamide and 0.5% phenylephrine hydrochloride eye drops. All procedures were performed under topical anesthesia. Patients in the low-energy FLACS group underwent femtosecond laser pretreatment with FEMTO LDV Z8 laser. The Z8 is a mobile femtosecond laser system that can be rolled into the operating theatre [13]. It performs fully automatic calibration with every start-up. The laser system allows for surgery to be performed without making any alterations to the operation room layout in terms of space and equipment. A disposable sterile patient liquid interface was applied to the eye centered over the limbus. The patient interface was filled with a balanced salt solution to create a liquid optic interface, then the handpiece attached to the articulating arm of the laser system was docked to the patient interface. The Z8 automatically monitors vacuum levels after docking, and immediately stops laser emission in case of loss of vacuum contact [20]. The handpiece is equipped with a color camera and optical coherence tomography (OCT) to image the ocular structures during cut positioning. Treatment parameters were customized to accommodate the differing eye and lens anatomy of each patient. Custom surgical planning software/algorithm identified ocular structures based on OCT images and automatically determined the suggested placement of surgical incisions and locations of lenticular cuts and associated safety margins. If needed, the surgeon had the ability to reposition treatment patterns via a touchscreen. After performing the femtosecond laser-assisted capsulotomy (5.2–5.3 mm diameter) and lens fragmentation (6 segments pie pattern) based on OCT-guided treatment mapping, the articulating arm of the mobile Z8 femtosecond laser was moved aside.

Further steps of the surgery were completed under the surgical microscope. Clear corneal incisions were made with standard corneal knives. The anterior chamber of the eye was filled with 1.7% sodium hyaluronate, a cohesive viscoelastic device (Shandong Bausch & Lomb Freda Pharmaceutical Co., Ltd.). Phacoemulsification was performed with the Centurion Vision System (Alcon Laboratories, Inc.). Patients in the CP group underwent manual continuous curvilinear capsulorhexis and lens fragmentation using a standard phacoemulsification technique that the surgeon performs regularly with the same Centurion phacoemulsification device. At each clinical center, the FLACS or CP surgery was completed by the same surgeon (one surgeon per center) to reduce the bias associated with differences in individual surgical technique. A monofocal aspheric IOL with a 6 mm optical zone, available from various manufacturers (see Additional file 1: Table S1 for the list of monofocal IOLs used) was implanted into the capsular bag through the appropriately-sized incision. After surgery, patients in both groups were subjected to antibiotic, non-steroidal anti-inflammatory eye drops, combined antibiotic and cortico-steroid both as eye drops and ointment, and artificial tear eye drops (if required). The postoperative care regimen was also identical in both groups and included antibiotic eye drops prescribed for 1–2 weeks, non-steroidal anti-inflammatory eye drops for 4 weeks, combined antibiotic and cortico-steroid eye drops for 4 weeks, combined antibiotic and corticosteroid ointment for 1 week, and artificial tear eye drops for about 3 months (as needed). Patients were followed at 1 day, 7 ± 2 days, 1 month (30 ± 7 days) postoperatively, 3 months (90 ± 14 days) postoperatively, and parameters including endothelial cell density (ECD), central corneal thickness (CCT), cumulative dissipated energy (CDE) and visual acuities were assessed. ECD was measured using the Konan specular microscope (Konan Medical, Hyogo, Japan), and CCT was measured using Pentacam HR.

The primary efficacy outcome was to compare the ECL between the two groups at 3 months post-surgery to assess the non-inferiority of FLACS as compared to CP. ECL at different postoperative time points was defined as the change in ECD between the respective time points and baseline. The secondary objectives of the study were to compare the FLACS and CP groups for CDE, the difference between pre and postoperative CCT at day 7, months 1 and 3; postoperative uncorrected and corrected distance visual acuities (UDVA and CDVA, respectively) at 3 months and total surgery time (time in minutes from surgery start to end, that included the time spent on the femtosecond laser machine, time spent on the phacoemulsification and IOL implantation and the time gap between the two procedures). Safety evaluation included intra/postoperative complications between the two groups.

### Statistical analysis

All statistical analyses were performed using the Statistical Analysis System (SAS) version 9.4. Continuous variables were reported as mean, standard deviation, and 95% confidence interval (CI); and categorical variables were expressed as frequency and percentages. The distribution of continuous variables was assessed by measures of normality and graphical displays. For normally distributed data, means between the two groups were compared using an independent two-sample t-test, and for non-normally distributed data, the non-parametric Mann-Whitney U test (Wilcoxon signed rank sum test) was used. For comparing proportions, the Chi-squared test was used. Generalized linear regression models were used to estimate the ECL and change in CCT from baseline between the two groups using the baseline ECD/CCT and clinical center as covariates. Efficacy analyses were performed on all patients (N = 132) who were randomized and safety analyses on patients who received either of the treatments (FLACS or CP surgery, N = 126).

A non-inferiority trial approach to sample size and power calculations was used for the comparison of the two treatment groups. The test for non-inferiority was one-sided at 2.5% significance level, 90% power, with the standard deviation for the ECD at 3 months, assumed to be 250 based on the observed standard deviation in recent FLACS trials [11, 21, 22], and mean ECD as 325 based on recent CP literature [11, 22]. Therefore, a total number of 120 eyes of 120 patients (60 in each group) were required. To compensate for approximately 10% of participants not completing 3 months of follow-up, 66 patients were recruited in each group (132 total). To demonstrate the non-inferiority of FLACS, a non-inferiority margin of − 150 cells/mm^2^ for ECL was used to compare the two groups. If the two groups demonstrated non-inferiority, a test for superiority was performed.

## Results

**Fig. 1:**
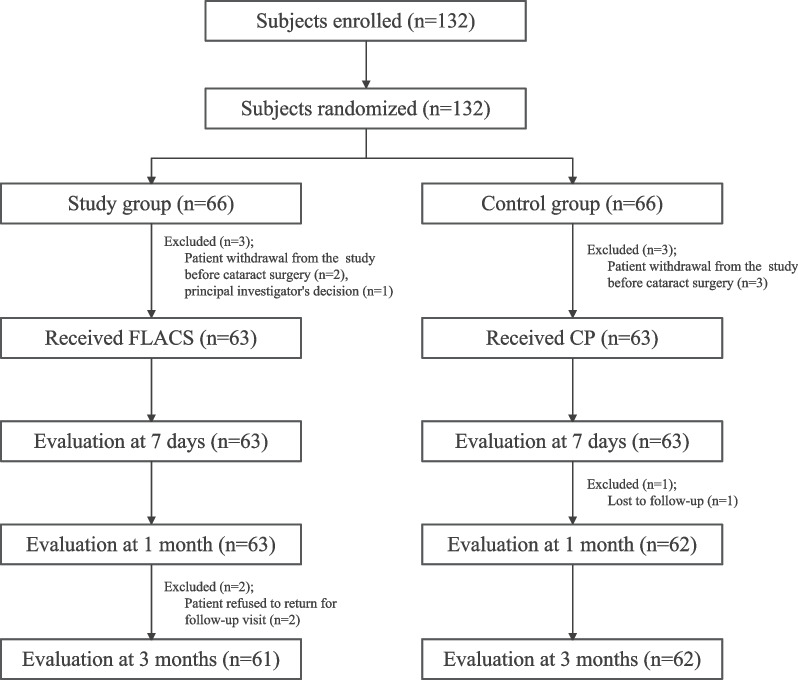
Subject disposition flow chart. FLACS, femtosecond laser-assisted cataract surgery; CP, conventional phacoemulsification

A total of 132 patients were enrolled and randomized in the study, 66 in the study group and 66 in the control group. Five patients (2 in the study group and 3 in the control group) withdrew from the study prior to the surgery, and one patient in the study group was excluded based on the investigator’s discretion prior to receiving treatment. Hence, a final total of 126 eyes of 126 patients received treatment (63 underwent FLACS and 63 underwent CP) (Fig. 1). The baseline demographic and ocular characteristics of patients were similar in both treatment groups (Table 1). There was no statistically significant difference in the preoperative cataract grade, mean axial length, anterior corneal power, endothelial cell density (ECD), and CCT between the FLACS and CP groups. The mean age of patients was comparable between the two groups (65.7 ± 6.3 versus 65.5 ± 6.8 years; *P* = 0.85).

**Table 1:** Demographics and baseline characteristics of study participants

Characteristics	Low-energy FLACS (n = 66)	Conventional phacoemulsification (n = 66)	*P* value
Age (years) (mean ± SD) range (min, max)			
	65.7 ± 6.3 (53, 79)	65.5 ± 6.8 (52, 79)	0.85
Gender n (%)			
Female	43 (65.2%)	43 (65.2%)	1.00
Male	23 (34.8%)	23 (34.8%)	
Race n (%)			
Han	66 (100.0%)	66 (100.0%)	
Nuclear opalescence (grade of cataract) n (%)			
1	21 (31.8%)	21 (31.8%)	0.79
2	36 (54.5%)	36 (54.5%)	
3	9 (13.6%)	8 (12.1%)	
4	0 (0.0%)	1 (1.5%)	
Cortical (grade of cataract) n (%)			
1	11 (16.7%)	9 (13.6%)	0.76
2	36 (54.5%)	33 (50.0%)	
3	14 (21.2%)	16 (24.2%)	
4	5 (7.6%)	8 (12.1%)	
Posterior subcapsular (grade of cataract) n (%)			
0	11 (16.7%)	9 (13.6%)	0.52
1	22 (33.3%)	20 (30.3%)	
2	13 (19.7%)	21 (31.8%)	
3	14 (21.2%)	13 (19.7%)	
4	6 (9.1%)	3 (4.5%)	
Axial length (mm) (mean ± SD) range (min, max)			
	23.7 ± 1.1 (22.0, 27.8)	23.5 ± 1.0 (21.2, 26.6)	0.24
Pupil diameter (mm) (mean ± SD) range (min, max)			
	2.70 ± 0.56 (1.82, 5.80)	2.84 ± 1.18 (1.53, 10.90)	0.39
Anterior chamber depth (mm) (mean ± SD) range (min, max)			
	2.71 ± 0.37 (1.95, 3.73)	2.71 ± 0.39 (1.72, 3.64)	1.00
Anterior mean corneal power (diopters) (mean ± SD) range (min, max)			
	43.8 ± 1.7 (38.8, 47.7)	44.3 ± 1.6 (40.8, 48.7)	0.08
Endothelial cell density (cells/mm^2^) (mean ± SD) range (min, max)			
	2647.0 ± 370.2 (1266.0, 3289.0)	2616.6 ± 340.9 (1773.0, 3472.0)	0.62
Central corneal thickness (µm) (mean ± SD) range (min, max)			
	540.6 ± 30.8 (472.0, 608.0)	534.3 ± 25.3 (464.0, 593.0)	0.20

FLACS = femtosecond laser-assisted cataract surgery; SD = standard deviation

### Primary outcomes

**Fig. 2:**
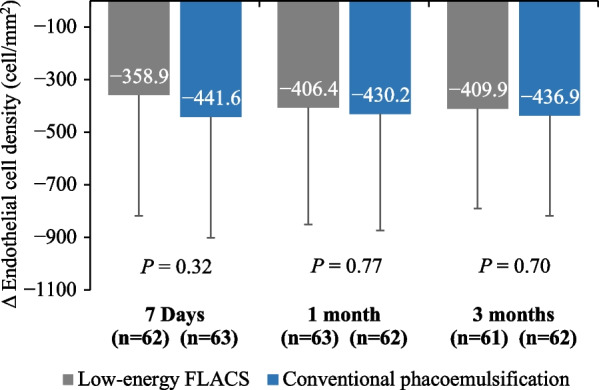
Changes in endothelial cell densities (ECD, cells/mm^2^) (adjusted for the preoperative values) in the femtosecond laser-assisted cataract surgery (FLACS) group and the conventional phacoemulsification group at different follow-up time points

The adjusted mean difference (95% CI) for ECL at 3 months (primary endpoint) was 27.0 cells/mm^2^ (− 109 to 163 cells/mm^2^). Since the lower bound of the 95% CI (− 109 cells/mm^2^) was greater than the non-inferiority margin of − 150 cells/mm^2^, the FLACS group was found to be non-inferior to the CP group. Superiority testing showed lower ECL in the FLACS group compared with the CP group, however, the difference was not statistically significant for the mean ECL at 7 days (95% CI: − 80.2 to 245.7, *P* = 0.32), 1 month (95% CI: − 133.8 to 181.5, *P* = 0.77) and 3 months (*P* = 0.70) in both treatment groups after adjusting for baseline ECD and clinical center (Fig. 2).

### Secondary outcomes

**Fig. 3:**
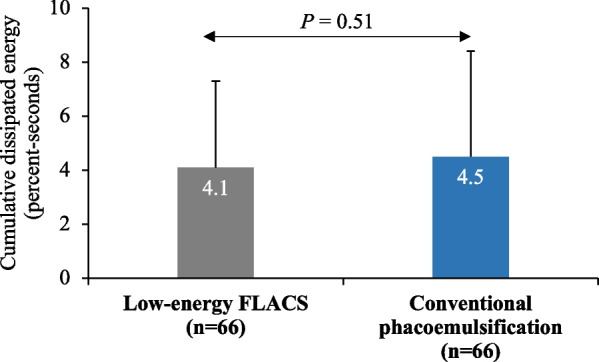
Values of cumulative dissipated energy (CDE) in the femtosecond laser-assisted cataract surgery (FLACS) group and the conventional phacoemulsification group

The mean CDE was also lower in the FLACS group as compared to the CP group, however, the difference was not statistically significant (*P* = 0.51; Fig. 3).

**Fig. 4:**
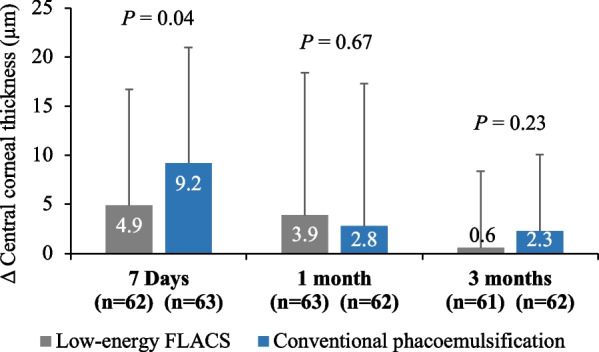
Changes in central corneal thickness (CCT, µm) (adjusted for the preoperative values) in the femtosecond laser-assisted cataract surgery (FLACS) group and the conventional phacoemulsification group at different follow-up time points

After adjusting for the baseline CCT and clinical center, the FLACS group showed a statistically significantly smaller increase in mean CCT on Day 7 than the CP group (4.9 versus 9.2 µm). The mean adjusted difference in CCT between the two groups was − 4.3 µm (95% CI: − 8.5 to − 0.2, *P* = 0.04) at Day 7, 1.12 µm (− 4.03, 6.28, *P* = 0.67) at 1 month, and − 1.69 µm (− 4.47, 1.09, *P* = 0.23) at 3 months (Fig. 4).

The mean postoperative UDVA and CDVA were comparable between the two groups with no statistically significant difference at any of the follow-up time points (all *P* ≥ 0.5).

The total surgery time was 14.2 ± 7.6 min (5–45 min) and 10.8 ± 7.8 min (5–41 min) in the FLACS and the CP group, respectively. The CP group showed shorter surgery time than the FLACS group (95% CI: 0.68–6.12, *P* = 0.015).

### Safety

No intraoperative complications were observed in either groups. A total of 8 postoperative complications occurred in 6 (9.5%) patients in the FLACS group and 9 postoperative complications occurred in 5 (7.9%) patients of the CP group, however, there was no significant difference in the rate of postoperative complications/adverse events between two treatment groups (*P* > 1.00). None of these complications in the FLACS group as well as the CP group were found to be related to the respective devices. Regarding reported complications, 2 complications (dryness and foreign body sensation) in the FLACS group and 4 complications (itching, posterior vitreous detachment, and corneal edema) in the CP group were classified as possibly related to the device.

The incidence of serious adverse events (SAEs) was comparable (*P* > 1.00) between the two groups; a total of 2 SAEs occurred in 2 (3.2%) patients in the FLACS group and 2 SAEs occurred in 1 (1.6%) patient of the CP group. All SAEs were non-ocular (cerebro-/cardio-vascular event, neurosensory deafness, and hypertension) and were not related to the surgery procedure or the device.

## Discussion

Several advantages of FLACS compared with CP have been documented in the literature [5, 7, 18, 23–26]. Lens fragmentation with femtosecond laser has been found to reduce phacoemulsification time/energy and decrease surgical manipulation in the anterior chamber. Capsulotomies created with femtosecond laser are precise, accurate, and reproducible in shape, centration, and dimensions, allowing for improved refractive outcomes due to a more predictable effective lens position. Most of the literature has researched high-energy femtosecond lasers; in contrast, our study evaluated the low-energy FEMTO LDV Z8 femtosecond laser and compared its safety and efficacy with CP surgery. Parameters including CDE, ECL, CCT, uncorrected and corrected visual acuity (UDVA and CDVA) were assessed.

CDE is a phacoemulsification parameter designed to monitor the amount of energy dissipated into the ocular tissues during phacoemulsification. Higher values of CDE are associated with longer surgery, more damage to the ocular tissue, and lengthier recovery times [27, 28]. Here, the mean CDE was found to be lower in the FLACS group compared with the CP group, although statistically not significant. Previously published studies have also reported lesser mean phacoemulsification time/energy in the FLACS group compared with the CP; while some studies reported this decrease to be statistically significant [4–6, 23, 29–32], others found no statistical differences between the techniques [18, 33, 34]. This incongruence in the results of different studies may be due to different patient populations, surgical techniques, and phacoemulsification devices. Future research in this regard may help decipher and better delineate factors responsible for this variation.

The FLACS group was also found to be non-inferior to CP in terms of preserving endothelial cell density. The lower bound of the 95% CI (− 109 cells/mm^2^) was greater than the non-inferiority margin of − 150 cells/mm^2^ in the FLACS group. At all postoperative time points (Day 7, Months 1 and 3), the FLACS group showed lower ECL compared with the CP group, although the difference did not reach statistical significance. This may be due to the high proportion of patients with grades 1 and 2 cataract in both groups. Higher mean ECL in the CP group can be attributed to the use of higher ultrasound energy that causes more cellular stress and damage to the corneal endothelium [35]. Further, ricocheting of nuclear fragments, fluid turbulence during irrigation/aspiration, and excessive anterior chamber manipulation may also lead to mechanical injury to the corneal endothelium resulting in ECL [36–38]. Laser pretreatment minimizes surgical manipulation required in the anterior chamber, decreasing damage to the collateral tissue, and is, therefore, less damaging to the corneal endothelium resulting in lower ECL [22].

Increase in CCT following cataract surgery is a metric to assess the functioning of corneal endothelium due to the surgical insult. It is affected not only by the mechanical/thermal injury-induced ECL but also due to the increased release of prostaglandins and associated postoperative inflammation. Our results were in line with experience from literature revealing a statistically significantly lower mean increase in the CCT at Day 7 in the FLACS group compared with the CP group. This trend continued through 1 month and 3 months after surgery with the FLACS group showing lesser increase in CCT compared with the CP group, although it did not reach statistical significance beyond Day 7. Statistically significantly lower increase in CCT at Day 7 in the low-energy FEMTO LDV Z8 group may be attributed to not only the lower CDE and lower ECL, but also decreased release of prostaglandins and resulting in lesser inflammation [39]. Low-energy femtosecond lasers have been shown to result in only a slight increase in prostaglandins levels compared with those reported with high-energy femtosecond laser systems [40, 41].

Visual acuities, whether uncorrected and corrected, were found to be comparable between the two groups. No intraoperative complications were observed in either of the two groups. Regarding total surgery time, FLACS took approximately one-third longer (mean difference of 3.4 min) due to the additional time spent on the laser procedure. The time difference is still shorter than previous studies since the following phacoemulsification procedure could be performed without moving the patient’s bed from the laser area to the surgical microscope with FEMTO LDV Z8 platform [42, 43].

The study has a few limitations, including a small sample size and a short follow-up of 3 months. However, it benefits from being a well powered (90%) and prospective multicentered study. Further studies with higher patient volumes and longer follow-ups are required to better assess the clinical efficacy and safety of low-energy femtosecond laser as well as a cost-benefit analysis of the emerging FLACS compared with CP.

## Conclusion

In conclusion, cataract surgery performed with the low-energy FEMTO LDV Z8 femtosecond laser was found to be safe and effective. Low-energy FLACS was also found to be non-inferior to CP. However, the FLACS group showed a significantly slight increase in CCT at Day 7 compared to the CP group which may be clinically relevant.

## Supplementary Information

**Additional file 1: Table S1:** List of monofocal aspheric intraocular lenses implanted in patients undergoing cataract surgery.

## Abbreviations

CCT: Central corneal thickness
CDE: Cumulative dissipated energy
CDVA: Corrected distance visual acuity
CI: Confidence interval
CP: Conventional phacoemulsification
ECD: Endothelial cell density
ECL: Endothelial cell loss
FLACS: Femtosecond laser-assisted cataract surgery
OCT: Optical coherence tomography
SAEs: Serious adverse events
SAS: Statistical analysis system
UDVA: Uncorrected distance visual acuity
