# Supplementary material for: Safety and efficacy of cataract surgery performed with a low-energy femtosecond laser compared with conventional phacoemulsification in Chinese patients: a randomized clinical trial
Source: Eye Vis (Lond). 2023 Jul 2;10:31. doi: 10.1186/s40662-023-00347-0 (PMC10315025; doi:10.1186/s40662-023-00347-0)
Supplement: Supplementary file 1 — Additional file 1: Table S1: List of monofocal aspheric intraocular lenses implanted in patients undergoing cataract surgery. [file 40662_2023_347_MOESM1_ESM.docx]

**Additional file 1: Table S1:** List of monofocal aspheric intraocular lenses (IOLs) implanted in patients undergoing cataract surgery.

| Model | Manufacturer | Material | Type | Color | Optic diameter  (mm) | Overall size  (mm) |
| --- | --- | --- | --- | --- | --- | --- |
| Aqua Sense PAL | Aaren Scientific | Acrylic hydrophilic | 1-piece, monofocal, foldable | Clear | 6.0 | 12.5 |
| ASPIRA-aA/-aAY | HumanOptics | Acrylic hydrophilic | 1-piece, monofocal, foldable | Clear/Yellow | 6.0 | 12.5 |
| CT ASPHINA 509M | Zeiss | Acrylic hydrophilic with hydrophobic surface | 1-piece, monofocal, foldable | Clear | 6.0 | 11.0 |
| iSert 251 | Hoya Surgical Optics | Acrylic hydrophobic | 1-piece, monofocal, foldable | Yellow | 6.0 | 12.5 |
| Promin A1-UV | Eyebright (China) | Acrylic hydrophobic | 1-piece, monofocal, foldable | Clear | 6.0 | 13.0 |
| PCB00 | Johnson & Johnson Vision | Acrylic hydrophobic | 1-piece, monofocal, foldable | Clear | 6.0 | 13.0 |
| SN60WF | Alcon | Acrylic hydrophobic | 1-piece, monofocal, foldable | Yellow | 6.0 | 13.0 |
| SN6AT series | Alcon | Acrylic hydrophobic | 1-piece, toric monofocal, foldable | Yellow | 6.0 | 13.0 |
| ZCT series | Johnson & Johnson Vision | Acrylic hydrophobic | 1-piece, toric monofocal, foldable | Clear | 6.0 | 13.0 |
